# The suppression of apoptosis by *α*-herpesvirus

**DOI:** 10.1038/cddis.2017.139

**Published:** 2017-04-13

**Authors:** Yu You, An-Chun Cheng, Ming-Shu Wang, Ren-Yong Jia, Kun-Feng Sun, Qiao Yang, Ying Wu, Dekang Zhu, Shun Chen, Ma-Feng Liu, Xin-Xin Zhao, Xiao-Yue Chen

**Affiliations:** 1 Institute of Preventive Veterinary Medicine, Sichuan Agricultural University, Wenjiang, Chengdu City 611130, Sichuan, P.R. China; 2 Key Laboratory of Animal Disease and Human Health of Sichuan Province, Sichuan Agricultural University, Wenjiang, Chengdu City 611130, Sichuan, P.R. China; 3 Avian Disease Research Center, College of Veterinary Medicine, Sichuan Agricultural University, Wenjiang, Chengdu City 611130, Sichuan, P.R. China

## Abstract

Apoptosis, an important innate immune mechanism that eliminates pathogen-infected cells, is primarily triggered by two signalling pathways: the death receptor pathway and the mitochondria-mediated pathway. However, many viruses have evolved various strategies to suppress apoptosis by encoding anti-apoptotic factors or regulating apoptotic signalling pathways, which promote viral propagation and evasion of the host defence. During its life cycle, *α*-herpesvirus utilizes an elegant multifarious anti-apoptotic strategy to suppress programmed cell death. This progress article primarily focuses on the current understanding of the apoptosis-inhibition mechanisms of *α*-herpesvirus anti-apoptotic genes and their expression products and discusses future directions, including how the anti-apoptotic function of herpesvirus could be targeted therapeutically.

## Facts

- Apoptosis plays an important and critical role in host defence mechanisms against viral infection.
- Viruses, including *α*-herpesviruses, encode anti-apoptotic virulence factors to evade elimination via apoptosis and to guarantee viral replication and propagation.
- Multiple signalling pathways, including the intrinsic and extrinsic pathways, form a complicated network to modulate apoptosis.
- Different viral anti-apoptotic genes have distinct mechanisms of mediating apoptosis.

## Open Questions

- In other evolving immune pathways, such as the autophagy and NF-*κ*B signalling pathways, what is the role of viral anti-apoptotic genes, and how do they interact with established protein networks?
- Can we properly exploit viral anti-apoptotic proteins as targets for antiviral drugs?

## Herpesvirus

*α*-Herpesviruses are responsible for severe morbidity and mortality, and they are characterized by a complex enveloped virion and a double-stranded DNA genome. They typically lyse productively infected cells and establish a reactivatable, latent infection in their hosts. Furthermore, *α*-herpesviruses have a relatively broad host range.^1^ The human *α*-herpesviruses include herpes simplex virus types 1 and 2 (HSV-1 and HSV-2) and varicella zoster virus (VZV). Moreover, there are several veterinary *α*-herpesviruses, including bovine herpesvirus (BHV), pseudorabies virus (PRV) and duck plague virus.

### Structure

**Figure 1:**
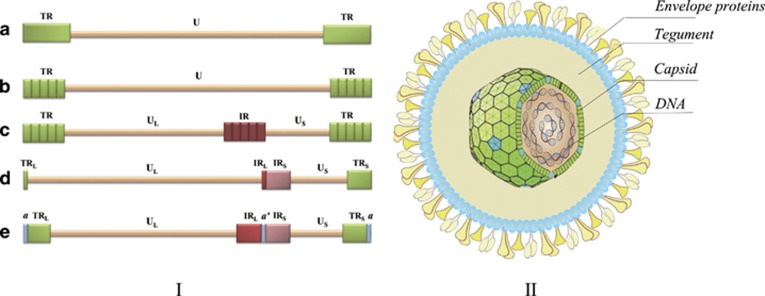
Overview of the types of herpesvirus genomes and the structure of virion. (I) The type A genomes (e.g., EHV-2) consist of a unique (U) region that is flanked by a direct terminal repeat (TR). The type B structure (e.g., Kaposi's sarcoma-associated herpesvirus) also consists of a U region flanked by variable numbers of TRs. The type C genomes (e.g., Epstein-Barr virus) harbour variable numbers of terminal sequences and an internal direct repeat that is unrelated to the TR and splits the U region into two unique regions (U_L_ and U_s_). The type D (e.g., PRV) and E (e.g., HSV-1) genomes contain U_L_ and U_S_ regions that are each flanked by terminal and internal inverted repeats (TR_L_/IR_L_ and IR_S_/TR_S_). The TR_L_/IR_L_ regions are very short in some type D genomes (e.g., VZV), whereas they are longer in viruses with class E genomes. In the type E structure, there is also a terminal direct repeat of hundreds of base pairs that is known as the *a* sequence. Moreover, an inverted copy, known as the *a'* sequence, is present internally. (II) The structure consists of four elements: (1) a core containing the viral dsDNA; (2) a T=16 icosahedral capsid encircling the core; (3) an amorphous protein layer called the tegument that surrounds the capsid; and (4) an outer lipid envelope (some inspiration came from these articles^5, 6, 7, 9, 10^)

The *α*-herpesvirus genomes range in size from approximately 120  to 180 kb, and their G+C nucleotide content ranges from 32 to 75%.^2, 3, 4^ The herpesvirus genomes are specified as type A, B, C, D or E genomes (Figure 1).^5, 6, 7^ Morphologically, herpesvirus virions share the similar strategy (Figure 1). The diameter of the virion is approximately 150–200 nm. The capsid consists of 162 capsomeres, containing 150 hexons of one protein species, 11 pentons of another protein and one portal vertex. There are three mature capsid forms A, B and C, which represent empty capsids, scaffold-containing capsids and viral DNA-containing capsids, respectively.^8^ The lipid envelope is derived from host cellular membranes and contains several viral membrane glycoproteins.^5, 9^ The glycoproteins have roles in adsorption, binding to the cell surface, and promoting fusion between the virion envelope and the cellular membrane.^10^

### Life cycle

The three subfamilies of herpesviruses share many strategies for replication. The two characteristic replication modes include a rapid, productive replication cycle and a life-long quiescent infection.

#### The lytic replication cycle

**Figure 2:**
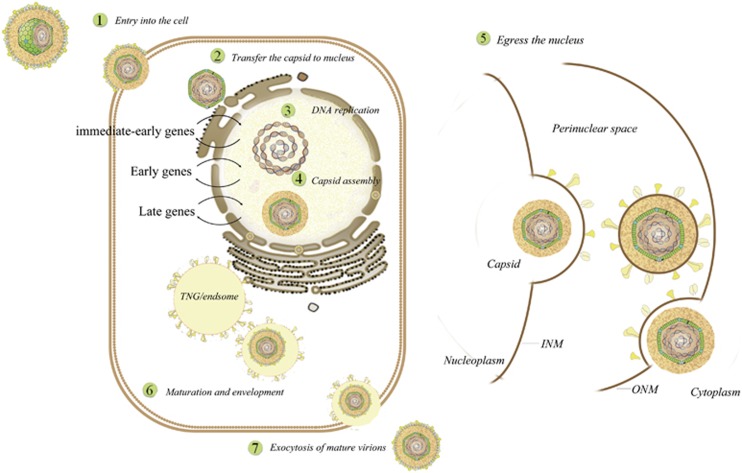
The lytic replication cycle of herpesviruses: (1) entry into the cell, (2) transfer of the capsids to the nucleus, (3) viral DNA replication, (4) capsid assembly, (5) egress from the nucleus, (6) maturation and envelopment of viral particles in the cytoplasm and (7) exocytosis of mature virions (some inspiration came from these articles^11, 12, 13, 14, 20, 23^)

Understanding of the lytic replication cycle comes primarily from studies of HSV-1 and PRV.^11, 12, 13^ Consequently, this cycle includes (1) entry into the cell, (2) the transfer of the capsids to the nucleus and viral DNA replication, (3) capsid assembly and egress from the nucleus, (4) the maturation and envelopment of viral particles in the cytoplasm and (5) the exocytosis of mature virions (Figure 2).

The *α*-herpesviruses attach to and then enter the cells of exposed mucosal surfaces to initiate infection. Depending on the cell type, virion entry can occur at either the plasma membrane or via endosomes after endocytosis.^14, 15^ In the primary mechanism, viral surface glycoproteins interact with cell-specific receptors to promote membrane fusion. Some of glycoproteins are involved in regulating apoptosis.^16, 17^

Upon fusion between the viral envelope and the cytomembrane, unenveloped virions enter the cytoplasm. Subsequently, capsids are transferred via microtubules to nuclear pores; motor proteins associated with microtubules are recruited through the exposure of inner tegument proteins, such as pUS3.^18^ Then, the viral genomes enter the nucleus through the nuclear pores and replicate. Herpesvirus gene expression can be divided into three types: immediate-early (IE or *α*), early (E or *β*) and late (L or *γ*). Functionally, IE genes, including *ICP0*, *ICP4*, *ICP22* and *ICP27*, are the first genes transcribed; the transcription is stimulated by the viral tegument protein VP16 and uses the host transcriptional apparatus. The E gene-encoded proteins, independently of viral DNA replication, can be activated by IE gene expression. Based on the strictness of the DNA replication requirement, L genes are subdivided into leaky (*γ*_1_) late and true late (*γ*_2_).^12, 19^ Additionally, herpesviruses also produce some non-coding RNAs and microRNAs that regulate the stability of viral and cellular mRNAs.

Subsequently, the DNA circularizes rapidly, and replication proceeds though a rolling circle mechanism. After expressing capsid proteins, herpesviruses assemble capsids and package their genomes into them. Unlike other DNA viruses, herpesviruses cannot obliterate nuclear membranes for their release.^20^ The viral capsids are too large (at least 120 nm) to pass through nuclear pores. Therefore, for egress, capsids are wrapped in the inner nuclear membrane (INM) and bud into the perinuclear space where they form primary enveloped capsids. During this process, the *α*-herpesvirus kinase pUS3 can regulate capsid nuclear egress by phosphorylating lamin A/C, which composes the nuclear lamina.^21^ Next, the perinuclear-enveloped capsids fuse with the outer nuclear membrane (ONM), and unenveloped capsids are delivered into the cytoplasm in a process called de-envelopment. Although gD is required for fusion during viral entry and is found in the perinuclear particles at the INM/ONM, gD deletion has no detectable effect on egress.^22^

A few tegument proteins, such as VP16 and VP22, are packaged in the nucleus, and others are added in the cytoplasm. In the cytoplasm, tegument-coated capsids are budded into specialized vesicles derived from the Golgi and endosomes. In this secondary envelopment process, not only are mature viruses produced but also the transport vesicles that later fuse with the plasma membrane to release the viruses.^23^ In secondary envelopment, the assembly of tegument and envelope is a complex process that involves numerous protein-protein interactions.^20, 23^ Enveloped virions, packaged into transport vesicles, can promote cell-to-cell spread or transport into neuronal axons for transmission to peripheral tissues.

#### Latency and reactivation

Importantly, all *α*-herpesviruses establish a latent infection in the neurons of the peripheral nervous system, which establishes a high-density viral reservoir and evades host antiviral defences.^24, 25, 26^ After viral replication at the portal of entry, *α*-herpesviruses concurrently invade the nervous system via fusion with neuronal membranes at axonal termini. Once the viral DNA has entered the nuclei in a ganglion, *α*-herpesviruses are immediately silenced and assembled into a chromatin structure resembling heterochromatin, which favours virus survival and establishes latent infection.^27^ In latently infected neurons, latency-associated transcript (LAT), which can silence lytic gene expression and block apoptosis, is the only gene expressed in high abundance.^28^

However, these mechanisms have been studied primarily with HSV-1. Different stimuli, such as fever, UV light and menstruation, can reactivate *α*-herpesviruses. Biologically, *α*-herpesviruses are reactivated by one of the following two conditions: (a) infection with a high viral genome load to overcome repression or (b) the translocation of key tegument proteins, such as VP16, to the nucleus where it derepresses viral DNA.^27^ Once reactivation has occurred, viral capsids are transported to the distal axon, where virions are assembled.^11, 29^

## Apoptosis

Apoptosis, also called programmed cell death, is a process that is common to all multicellular organisms for eliminating cells via a complex but highly defined programme. Apoptotic cells are characterized by nuclear fragmentation, cell shrinkage and the formation of apoptotic bodies.^30^ Virus-induced apoptosis can limit viral replication and transmission. Although apoptosis contributes to the prevention of pathogenesis, it is a potentially costly and even vainly attempted self-sacrifice.^31, 32^

Notably, viruses have evolved anti-apoptotic mechanisms to maintain their replication and propagation.^33^ To escape elimination via apoptosis, viruses target and regulate key regulatory steps in the apoptotic pathway; they can inhibit death receptor-mediated apoptotic signals, regulate mitochondrial permeability and suppress the caspase cascade.^34, 35, 36^ Moreover, viruses can inhibit apoptosis by regulating the pro-apoptotic tumour suppressor p53.^37^

*α*-Herpesvirus can encode anti-apoptotic viral proteins, such as US3, gJ and LAT, to interfere with the apoptosis pathway.^36^ Understanding the mechanisms by which *α*-herpesvirus regulates apoptosis is useful for future research. In this article, we focus on reviewing the roles played by *α*-herpesvirus proteins in suppressing apoptosis.

### The apoptotic pathway

Apoptosis is modulated by two signalling pathways – the intrinsic and the extrinsic pathways. The extrinsic pathway, also called the death receptor pathway, is regulated by membrane death receptors, which are activated by binding between a ligand and its receptor.^38^ Conversely, the intrinsic pathway is modulated by intracellular stimuli, such as oxidative stress, hypoxia and nutrient deprivation, which lead to mitochondrial outer membrane permeabilization (MOMP). Subsequently, cytochrome *C* is released into the cytoplasm.^39, 40^ Both pathways can activate the enzymatic cysteine-specific aspartate protease (caspase) cascade, triggering numerous proteolytic events that mediate the apoptotic cell death programme.^40^ The underlying mechanisms of apoptosis are now being unravelled. We will review the intrinsic and extrinsic signalling pathways to better understand the mechanisms by which *α*-herpesvirus perturbs apoptosis.

#### The extrinsic pathway

**Figure 3:**
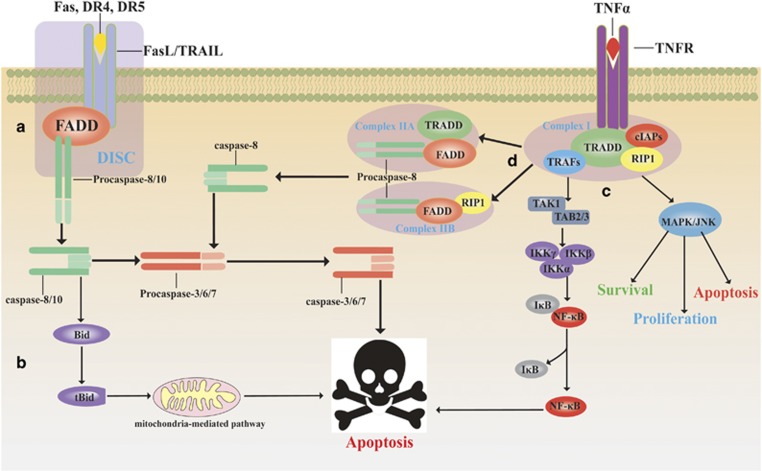
The extrinsic apoptosis pathway. (a) Fas and DR4/5 are activated by the binding of their respective ligands FasL and TRAIL; the receptors then bind to FADD via the death domain (DD). Then, the death effector domain (DED) of FADD binds to procaspase-8/10, forming the death-inducing signalling complex (DISC) to facilitate the autoproteolytic cleavage of procaspase-8/10, which induces the activation of the caspase cascade and ultimately results in apoptosis. (b) In particular cells, activated caspase-8 cleaves the pro-apoptotic protein Bid to create truncated Bid (tBid), and this results in the activation of the mitochondria-mediated apoptotic signalling pathway. (c) TNFR1, EDAR and DR3/6 are activated by the binding of their respective ligands. TNFR1 recruits TRADD, an adaptor protein that binds to TNF receptor-associated factors (TRAFs), receptor-interacting protein kinase (RIP1) and cellular inhibitor of apoptosis (cIAPs), forming the initial membrane pro-survival complex (complex I), which stimulates the MAPK/JNK and NF-*κ*B pathways to facilitate cell survival or apoptosis. (d) Complex I forms two types of cytoplasmic apoptotic complexes, TRADD-dependent complex IIA and RIP1-dependent complex IIB, which activate caspase-8, thus initiating apoptosis (some inspiration came from these articles^47, 48, 49, 50, 51, 52^)

The extrinsic apoptotic pathway is a process in which specific ligands or apoptotic signals bind to membrane death receptors (DRs) to initiate apoptosis (Figure 3). These DRs belong to the TNF superfamily. To date, the eight TNF superfamily receptors CDl20a (p55/TNFR1), CDl20b (TNFR2), CD95 (Fas/ApoI), DR3, DR4 (TRAILR1), DR5 (TRAILR2), NGFR and EDAR have been identified in this process.^41^ Fas and TNFR1, the representative receptors, can bind with Fas ligand (FasL) and tumour necrosis factor-related apoptosis-inducing ligand (TRAIL), respectively, to form a death-inducing signalling complex (DISC) and activate a caspase cascade.^42, 43^

##### Mediated by FADD

Fas-associated protein with death domain (FADD), an apoptosis-related adaptor protein, is composed of a death domain (DD) and a death effector domain (DED).^44^ Fas, DR4 and DR5 are activated by specific ligands and bind to FADD through the DD domain. Then, the DED of FADD binds to procaspase-8/10 to construct the DISC. The DISC promotes the autoproteolytic cleavage of procaspase-8/10.^45^ The activation of caspase-8/10, which have enzymatic activity, can hydrolyse caspase-3/6/7 to induce apoptotic conditions, including cell shrinkage, nuclear fragmentation, apoptotic DNA fragmentation and ultimate cell death.^44, 46, 47, 48, 49^

##### Mediated by TRADD

TNFR1-associated death domain protein (TRADD), an adaptor molecule, transduces the signal downstream of TNFR1, and TRADD contains a DD. Other death receptors, including TNFR1, DR3, DR6 and EDAR, recruit TRADD, which links DRs to TRAF2 (TNF receptor-associated factor 2), RIP (receptor-interacting protein kinase) and cIAPs (cellular inhibitor of apoptosis), forming a signalling complex named complex I.^41, 49^ TRADD interacts with TRAF2 via its N-terminal TRAF2-binding domain and with RIP via the DD. The initial formation of the membrane pro-survival TRADD–RIP1–TRAF2 complex I is followed by the assembly of a cytoplasmic apoptotic complex (complex II). There are two types of cytosolic complex II. FADD and caspase-8 are recruited by TRADD to form TRADD-dependent complex IIA, and RIP1 recruits FADD and caspase-8 into complex IIB, which can be negatively regulated by cIAPs. Both complex IIA and IIB can initiate apoptosis by activating caspase-8.^44, 50, 51^ Additionally, complex I stimulates the mitogen-activated protein kinase/c-Jun N-terminal kinase (MAPK/JNK) pathway to induce cell survival, proliferation or apoptosis, and it modulates the nuclear factor kappa B (NF-*κ*B) pathway, which facilitates cell survival, inflammatory signalling and apoptosis.^50, 52^

### The intrinsic pathway

**Figure 4:**
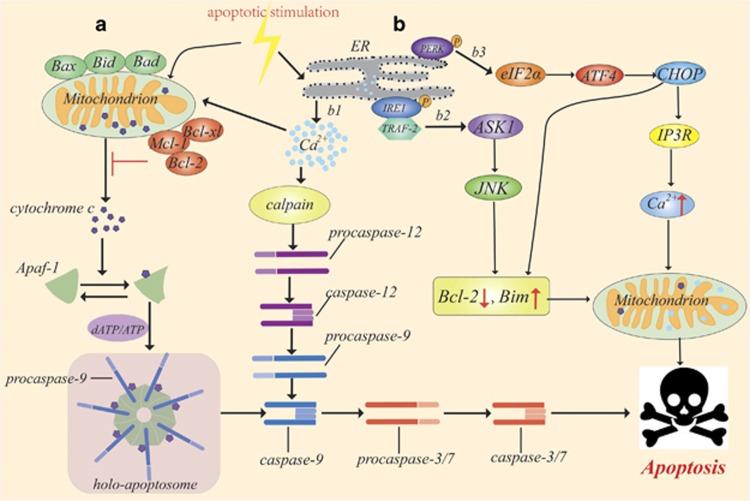
The intrinsic apoptosis pathway. The intrinsic apoptotic pathway is triggered by intracellular stimuli. (a) Intracellular apoptotic stimuli upregulate the pro-apoptotic Bcl-2 family of proteins, such as Bax, Bid, Bak and Bad, leading to the mitochondrial release of cytochrome *C*, which binds to Apaf-1. The replacement of ADP by dATP/ATP in Apaf-1 triggers the formation of a heptameric apoptosome, which assembles with procaspase-9 to form the holo-apoptosome. The Apaf-1 apoptosome catalyses the cleavage and activation of procaspase-9, which triggers the caspase cascade, and the activation of caspase-3 and caspase-7 leads to eventual apoptosis. (b) Under ER stress, three upstream signalling proteins – IRE1, PERK and ATF6 – are activated, thus leading to a cascade of activity that induces apoptosis. (b1) The ER releases Ca^2+^ from the ER lumen into the cytoplasm, which triggers apoptosis by activating the calcium-sensing kinase CaMKII. Then, CaMKII activates procaspase, which in turn triggers caspase cascade activation. (b2) The prolonged activation of IRE1 can promote apoptosis. Phosphorylated IRE1 recruits TRAF2 (TNF receptor-associated factor 2) and triggers a cascade of phosphorylation events, such as the activation of ASK1 (apoptosis signalling kinase 1), which ultimately phosphorylates and activates JNK. Then, JNK phosphorylation activates pro-apoptotic Bim and blocks anti-apoptotic Bcl-2. (b3) The homomultimerization and autophosphorylation of PERK leads to eIF-2*α* (eukaryotic translation initiation factor 2*α*) phosphorylation, which increases the translation of ATF4 (activating transcription factor-4). Then, ATF4 upregulates the expression of CHOP (C/EBP-homologous protein), which promotes apoptosis through two of the major cell death pathways – the IP3R–Ca^2+^–CaMKII pathway and the Bcl-2 family member pathway (some inspiration came from these articles^56, 57, 58, 59, 60, 61^)

The intrinsic pathway, or the mitochondria-mediated apoptotic pathway (Figure 4), is initiated by intracellular stimuli that trigger MOMP, which allows the release of cytochrome *C* from mitochondria and is closely regulated by the Bcl-2 family.^53^ Bcl-2 family proteins are divided into pro-apoptotic proteins (Bax, Bid, Bak, Bad) and anti-apoptotic proteins (Bcl-2, Bcl-xl, Mcl-1, A1). A slight change in the dynamic balance of these proteins leads to apoptosis or apoptosis inhibition.^54, 55, 56^

Pro-apoptotic Bcl-2 family members respond to a variety of intracellular apoptotic stimuli and then form pores in the outer mitochondrial membrane through which cytochrome *C*, which is normally bound to the inner mitochondrial membrane, is released into the cytoplasm.^57^ The released cytochrome *C* binds to Apaf-1 via *β*-propellers, and it then triggers nucleotide exchange and the gradual assembly of the apoptosome.^58^ The Apaf-1 and procaspase-9 caspase recruitment domains (CARDs) co-assemble to form a CARD–CARD disk, thus assembling the holo-apoptosome, which creates an asymmetric proteolysis machine. Each holo-apoptosome can recruit five to seven procaspase-9 molecules by interacting with the N-terminal CARDs of Apaf-1.^59^ The tethered catalytic domains of multiple procapase-9 molecules result in the activation of zymogen, which cleaves procaspase-9. Subsequently, caspase-9 triggers the caspase cascade, and then the activation of caspase-3/7 executes apoptosis.^53, 58^

Furthermore, chronic or unresolved endoplasmic reticulum (ER) stress, which can be caused by increased protein synthesis or misfolding rates and alterations in Ca^2+^ stores, is involved in the mitochondrial apoptotic pathway.^60^ Under ER stress, the ER-specific unfolded protein response is triggered through IRE1 (inositol requiring protein-1), PERK (protein kinase RNA-like ER kinase) and ATF6 (activating transcription factor-6) apoptotic pathways, which are connected to the mitochondria-mediated apoptotic pathway.^61^

## The Anti-apoptotic Genes of *α*-herpesvirus

To evade elimination, *α*-herpesviruses have gradually evolved various host immune modulation strategies to promote their fitness and pathogenesis. Although cells infected with viruses can be eliminated by cellular apoptosis, viruses can also suppress this apoptosis by encoding anti-apoptotic virulence factors. Here, we review the current understanding of the apoptosis-inhibiting mechanisms of the *α*-herpesvirus anti-apoptotic genes and their expression products (Table 1).

### LAT and its mechanism of apoptosis regulation

During the latent stage, LAT is the only highly transcribed viral gene. LAT enhances the establishment of latency, reactivates the virus from latency and protects the host cells from apoptosis.^62, 63^ Moreover, HSV-1 LAT can inhibit capase-8/9-mediated apoptosis stimulated through either the intrinsic or extrinsic pathway.^64, 65, 66, 67^

**Figure 5:**
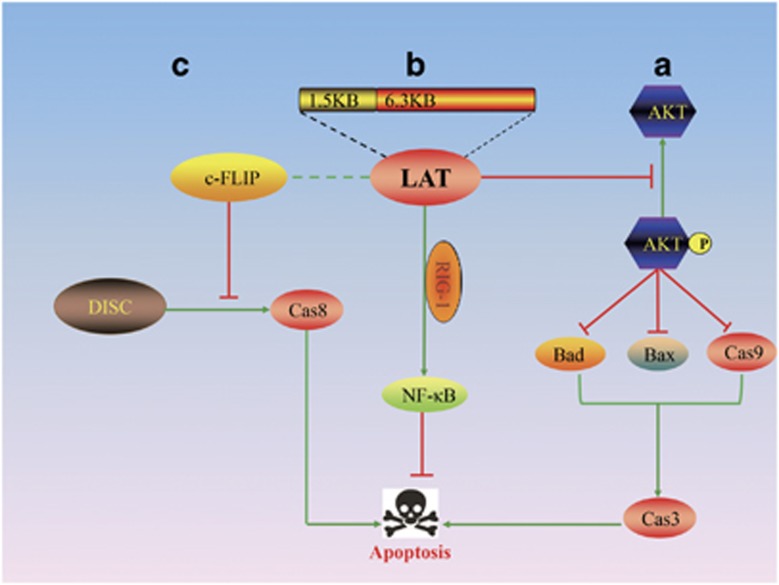
The anti-apoptotic pathway of LAT. The main domain of the LAT gene is contained in the first 1.5 kb. LAT can inhibit apoptosis by interacting with the following pathways: (a) LAT stabilizes the levels of total and phosphorylated AKT protein, which phosphorylates and inactivates the pro-apoptotic proteins Bad, Bax and caspase-9 to ultimately regulate caspase-3 activation and block apoptosis; (b) LAT cooperates with RIG-1 to promote NF-*κ*B-dependent transcription to interfere with apoptosis and benefit cell survival; and (c) LAT substitutes for the function of c-FLIP, which is an inhibitor of caspase-8-mediated apoptosis

The anti-apoptotic mechanisms of LAT are being unravelled (Figure 5). Stable cell lines expressing LAT can block caspase-3 activation, thus confirming that LAT can suppress apoptosis even in the absence of other HSV-1 genes.^28^ HSV-1 LAT can stabilize the levels of total and phosphorylated AKT protein, which phosphorylates and inactivates the pro-apoptotic proteins Bad, Bax and caspase-9, to regulate caspase-3 activation and ultimately block apoptosis.^68, 69^ In addition, a stable cell line expressing LAT can recover from cold-shock-induced apoptosis, which supports the notion that LAT can inhibit the dephosphorylation of pAKT and stabilize phosphorylated AKT.^70^ Furthermore, the inhibition of pAKT dephosphorylation by LAT promotes cell survival and virus latency by protecting against the cleavage of caspase-2/3/9 and increasing the ratio of Bcl-2/Bcl-xl (or Bcl-2/tBid).^70^

Additionally, LAT inhibits apoptosis by blocking GrB- and Fas-mediated caspase-3 cleavage, which protects Neuro2A and C1300 cells against cytotoxic CD8 T-cell-mediated death.^71^ The cellular FLICE-like inhibitory protein (c-FLIP), an inhibitor of caspase-8-mediated apoptosis, can substitute for LAT to enhance the reactivation phenotype.^72^ Furthermore, downregulating c-FLIP expression via small interfering RNA markedly promoted apoptosis in uninfected immature dendritic cells, and its function could be replaced by LAT.^73^ Thus, LAT either inhibits multiple steps in apoptotic cascades or regulates AKT and pAKT levels to promote cell survival.

HSV-1 LAT is an 8.3-kb gene, and splicing yields two forms – a stable 2-kb LAT and an unstable 6.3-kb LAT. The first 1.5 kb of the primary 8.3-kb LAT has anti-apoptotic activity,^66^ and it contains eight open reading frames (ORFs) based on sequence analysis. Point mutation of six ATGs in the intron reduces the anti-apoptotic effect on caspase-9 but not on caspase-8, whereas mutating the two ATGs in the exon does not completely eliminate the anti-apoptotic activity that affects caspase-8/9.^74^ The first 1.5 kb of LAT encodes two small RNAs (sRNA1 and sRNA2) of 62 nt and 36 nt, respectively, and they cooperate to block apoptosis.^75^ They also cooperate with RIG-1 to promote NF-*κ*B-dependent transcription to inhibit apoptosis and benefit cell survival.^76^

Notably, HSV-2 LAT also shows anti-apoptotic activity. ORF1, ORF2, and ORF3 of HSV-2 LAT protect Vero cells from apoptosis induced by actinomycin D (ActD), 5-FU and cisplatin, respectively,^77, 78, 79, 80^ but the underlying mechanism remains elusive. Similarly, BHV-1 latency-related RNA (LR-RNA) encodes two microRNAs and a protein (ORF2) that can protect cells from stress-induced apoptosis by stimulating NF *κ*B and activating AKT.^81^

### ICP22 and its mechanism of apoptosis regulation

Infection cell protein 22 (ICP22), an IE protein, is required for efficient replication in restrictive cells and the conventional expression of viral late proteins. HSV-1 US1 encodes the 420-amino-acid protein ICP22 and produces an in-frame, N-terminally truncated form of ICP22 called US1.5, which contains 273 amino acids.^48, 82^ US1.5 becomes detectable and accumulates only at a later time point after infection.^83^ It is generally believed that ICP22 not only promotes apoptosis but also inhibits apoptosis. The pro-apoptotic function is thought to be regulated by US1.5, which activates caspase-3.^83^

The ICP22-deleted HSV-1 induces more apoptotic cells, demonstrating that ICP22 can block apoptosis.^67^ Moreover, ActD-induced apoptosis can be blocked by transfecting a pEGFP-ICP22 plasmid.^84^ Nevertheless, the anti-apoptotic activity of ICP22 is not strong. Thus, ICP22-mediated anti-apoptosis is not likely acting directly on apoptotic signalling but instead precisely regulates the expression of viral early and late genes, including anti-apoptotic genes. For example, in the absence of ICP22, the expression of US5 is delayed.^85^

Additionally, cells infected with HSV-1 d120 (an apoptosis inducer), which blocks the ICP4 and US3 genes, show an additional product known as Mr 37 500, which results from the proteolytic cleavage of ICP22 by caspase-3. Furthermore, this process is suppressed by over-expressing Bcl-2, transfecting US3 or adding caspase-3 inhibitor.^86^ During viral infection, viral protein cleavage by caspases leads to a variety of consequences, such as the counteraction of apoptosis, the enhancement or weakening of replication and the spread of the virus.^87^ Furthermore, ICP22 can antagonize p53. p53 is a master factor in the cell that modulates various cellular pathways and controls apoptosis by activating Bax or inhibiting Bcl-2.^88^ p53 has dual effects on HSV-1 replication; it plays a positive role by enhancing ICP27 expression and a negative role by inhibiting ICP0 expression. The negative effect of p53 is suppressed through interaction with ICP22, which promotes cell survival and efficient replication.^89^ However, it remains unclear whether ICP22 can directly regulate apoptosis by interacting with p53.

VZV causes diseases of the skin or mucosa in a similar fashion as HSV-1. Moreover, VZV ORF63, which encodes the immediate-early protein 63 (IE63) and shares homology with HSV-1 ICP22, can also block apoptosis.^90, 91^ VZV ORF63-deleted VZV-infected human neurons demonstrate significantly increased apoptosis compared with neurons infected with the parental VZV. Furthermore, expressing ORF63 alone can inhibit apoptosis, showing that VZV can encode an anti-apoptotic gene.^92^ Compared with parental virus, ORF63-deleted VZV has higher levels of phosphorylated eIF-2*α* to promote apoptosis. Meanwhile, the expression of IE63 alone can be sufficient to block eIF-2*α* phosphorylation.^93^ Thus, like its homologue HSV-1 ICP22, VZV ORF63 can inhibit apoptosis, but it acts through exceedingly different mechanisms.

### ICP27 and its mechanism of apoptosis regulation

Infection cell protein 27 (ICP27) is an IE protein that is indispensable for viral replication. Interestingly, ICP27 is the only IE protein that has homologues in all of the herpesviruses. ICP27 is a multifunctional protein that performs various activities during the viral life cycle,^94, 95^ such as shutting down host protein synthesis,^96^ promoting viral DNA synthesis and expression,^97, 98^ orchestrating all stages of viral mRNA biogenesis,^99, 100^ activating stress signalling pathways^101^ and blocking apoptosis.^102^

Based on studies comparing an HSV-1 ICP27-deletion virus with a wild-type virus, ICP27 was shown to block apoptosis.^102, 103, 104^ Moreover, ICP27-null virus-induced apoptosis in HEp-2 cells is closely associated with the activation of caspase-3.^104^ A succession of viral ICP27 mutants revealed that encompassing 20–65 amino acids near the N-terminus are required for p38 and modest JNK signalling activation.^105, 106^ However, the C-terminus can indirectly inhibit apoptosis by increasing the expression of anti-apoptotic genes, such as, gJ and gD.^85, 105, 107^

Moreover, expressing ICP27 can directly activate p38 signalling and partially activate JNK signalling.^108^ Additionally, ICP27-mediated JNK signalling during HSV-1 infection induces the activation of NF-*κ*B, which is associated with the inhibition of apoptosis.^109, 110^ Interestingly, the region from amino acids 21 to 63 is also required for I*κ*B*α* stabilization.^109^ ICP27 suppresses the phosphorylation and ubiquitination of I*κ*B*α*, thus stabilizing I*κ*B*α* and inhibiting NF-*κ*B activity, which progressively blocks apoptosis.^101^

Furthermore, as a multifunctional protein, ICP27 is not only an inhibitor of apoptosis but is also capable of triggering cell death. Using chemical inhibitors, it was shown that the ICP27-mediated activation of p38 signalling can stimulate the apoptotic pathways.^108^ However, during a viral infection, p38 also mediates the destabilization of Bcl-2 to prevent apoptosis; this process also requires ICP27 expression.^111^ ICP27 might alternate between pro- and anti-apoptotic activity by changing the Bax/Bcl-2 ratio in the course of reactivation from a latent state.^112^ To summarize, ICP27 is an important regulator of host cell fate. It promotes maximum viral replication in the early phase of infection and greatly enhances viral release in the late stage.

### US3 and its mechanism of apoptosis regulation

*US3* is represented by ORF3 in the U_S_ region of the herpesvirus genome, and it encodes a serine/threonine protein kinase that is highly conserved throughout the *α*-herpesvirus subfamily. *US3*-encoded proteins have an ATP binding domain and a catalytic active site in the kinase domain.^113^ Although the *US3* protein kinase (PK) is not required for viral replication, it can regulate the biological functions of the virus and the host cells. For example, *US3* PK is involved in the egress of nucleocapsids,^114^ the maturation of virions,^21, 115^ enhancing viral spread,^116^ rearranging the actin cytoskeleton^117^ and evading antiviral responses.^118^ *US3* PK can block apoptosis induced by viral infection, the overexpression of Bcl-2 family proteins, cytochrome *C* release and Fas/UV-mediated apoptosis.^119, 120, 121, 122, 123^

#### US3 regulation of the intrinsic pathway

Expressing *US3* suppresses the release of cytochrome *C* and the activation of procaspase-3 in HEp-2 cells infected with HSV-1 d120 mutant, thus blocking apoptosis at the pre-mitochondrial stage.^124, 125^ Furthermore, active *US3* PK also phosphorylates procaspase-3 to enhance apoptosis resistance.^125^ These studies indicate that *US3* PK may target specific molecules in the cellular pro-apoptotic pathway to block apoptosis and promote viral replication.

*US3* PK also inhibits apoptosis induced by the overexpression of Bad and Bid, which are parallel signalling proteins in the mitochondria-mediated apoptotic pathway. Thus, *US3* also prevents apoptosis by affecting downstream effectors of the mitochondria-mediated pathway.^122, 126^ Specifically, *US3* blocks apoptosis by phosphorylating Bad and inactivating caspases that cleave Bad to render the protein more pro-apoptotic.^123^ PRV *US3* prevents apoptosis with the same efficiency as HSV-1 *US3*.^127^ Point mutation in the putative ATP binding site of the PRV *US3* gene removes its ability to resist apoptosis, and it does not phosphorylate pre-apoptotic Bad.^128^ Furthermore, transiently transfected PRV *US3* can induce Bad serine-112 and serine-136 phosphorylation, the downregulation of pro-apoptotic Bax, and the upregulation of anti-apoptotic Bcl-2 to regulate apoptosis.^129^ Both HSV-1 *US3* and PRV *US3* can prevent apoptosis induced by Bcl-2 family proteins, demonstrating the conserved anti-apoptotic role of *US3*.^122^

#### US3 regulation of the extrinsic pathway

Accordingly, *α*-herpesvirus-encoded proteins such as *US3* PK and ICP27 can modulate the NF-*κ*B signalling pathway, which can mediate cytokine production and virus-driven apoptosis.^101, 130^ Furthermore, the kinase activity of *US3* can inhibit NF-*κ*B activation and NF-*κ*B-mediated cytokine production.^131, 132^ The NF-*κ*B signalling pathway can be activated by TLR-dependent signalling. Additionally, *US3* can block TLR2 signalling by interacting with proteins downstream of MyD88 and upstream of p65, and it can reduce TRAF6 polyubiquitination.^131^ Moreover, *US3* interacts with endogenous p65 to lead to the hyperphosphorylation of p65 at serine 75, which sufficiently prevents the translocation of p65 and blocks the nuclear accumulation of NF-*κ*B.^132^

Likewise, PRV *US3* PK also regulates the NF-*κ*B pathway to suppress apoptosis. *US3a* and *US3b*, two isoforms of PRV *US3*, both upregulate anti-apoptotic signalling through transient overexpression, but the anti-apoptosis effect of *US3a* is greater than that of *US3b*. Similar to HSV-1 *US3*, the kinase activity of PRV *US3* is indispensable for its anti-apoptotic activity.^129^ In the presence of *US3*, phosphorylated I*κ*B*α* increases in the early stage of infection to suppress apoptosis. In addition, transiently transfecting *US3a* or *US3b* can produce more phosphorylated AKT, an anti-apoptotic protein, and its activator molecule PDK-1, which is responsible for the PI3K-dependent phosphorylation of AKT serine 473. Comparatively, HSV-1 *US3* can activate AKT substrate phosphorylation by targeting the downstream AKT signalling molecules.^133^ HSV-1 *US3* masquerades as AKT, even though it does not look like AKT, and it phosphorylates tuberous sclerosis complex 2 (TSC2) on the same residues phosphorylated by AKT (T1462/S939^134^), indicating that phylogenetically related viruses have different mechanisms of modulating the same signalling pathways.

Additionally, *US3* can bind and phosphorylate group A p21-activated kinases (PAKs), thus regulating Cdc42/Rac GTPase signalling, which is involved in *US3*-mediated cytoskeletal rearrangements.^135^ Interestingly, activated PAK1 phosphorylates the pro-apoptotic protein Bad, and activated PAK2 also regulates the activity of Bad.^136, 137, 138^ Investigating the involvement between PAKs and *US3* showed that PAK2 has no effect on *US3*-mediated anti-apoptosis, whereas PAK1 has a significant, yet limited, effect. Interestingly, human immunodeficiency virus (HIV)-associated anti-apoptotic protein targets PAKs to induce anti-apoptotic signalling. This mechanism is similar to that of the *US3* PK of *α*-herpesvirus,^139^ indicating that viruses of different genera share some common strategies to evade host immunity.

cAMP-dependent protein kinase (PKA) is similar to the substrate specificity of *US3* PK, which can regulate apoptosis.^140^ *US3* can phosphorylate the regulatory subunit of PKA, such as the autophosphorylation site of RII*α*, to promote a redundancy of anti-apoptotic events.^140, 141^ In addition, programmed cell death protein 4 (PDCD4), a tumour suppressor, interacts with HSV-1 *US3* PK to regulate apoptosis.^142^

### Viral glycoproteins and their apoptosis regulation mechanisms

Herpesvirus glycoproteins are critical for viral entry and for transmission between host cells, and they can undermine host defences, including apoptosis, thus helping viruses persist for a lifetime.^143^ Some viral glycoproteins, such as gD, gE and gJ, have been shown to play roles in the suppression of apoptosis.

*US5*, a poorly conserved gene, is represented by ORF5 in the US region; it is a late gene that encodes glycoprotein J and is unnecessary for viral replication. Thus, the *US5* gene of herpesvirus has been much less carefully studied, except with respect to its function of blocking apoptosis. The deletion of US5 from HSV-1 markedly abrogated protection from Fas-mediated apoptosis and partially reduced the protection from UV radiation by suppressing the activation of caspase-3/8.^120^ Transfecting *US5* inhibits Fas/UV-induced apoptosis and weakens the activation of caspases.^144^ In sum, gJ is sufficient to establish an anti-apoptotic phenotype. *US5*-deleted HSV-1 shows weakened protection from cytotoxic T lymphocyte-induced apoptosis, and gJ is sufficient to inhibit F_0_F_1_ ATP synthase activity and reactive oxygen species creation to suppress apoptosis.^85, 145^ However, the mechanism by which gJ blocks apoptosis remains elusive.

*US6*, encoding glycoprotein D, is represented by ORF6 in the US region. *US6* is the main component of the envelope and is essential for virus dissemination. gD of HSV-1 and BHV-1 can inhibit apoptosis.^146, 147, 148^ Compared with gJ, gD blocks apoptosis at different stages of the viral life cycle.^17^ The binding of HSV-1 gD to herpesvirus entry mediators, such as mannose-6 phosphate receptor, can suppress premature apoptosis, distinguishing gD from other anti-apoptotic genes.^146, 149^ Additionally, gD can inhibit Fas-mediated apoptosis, which involves the activation of NF *κ*B.^150^ Furthermore, with the inactivation of NF *κ*B, HSV-1-driven apoptosis increases and is accompanied by high gD expression.^151^ However, the molecular signalling involved in the anti-apoptotic activity of gD remains poorly understood.

*US8* is represented by ORF8 in the US region and encodes glycoprotein E, which is also a late gene (*γ*) and an important *α*-herpesvirus virulence factor. PRV gE activated ERK1/2 in T lymphocytes and epithelial cells, and this was associated with the degradation of the pro-apoptotic protein Bim.^152^ Notably, this is the first time that gE was associated with the apoptosis signalling pathway.

## Future Perspective

Viruses must evade host defence mechanisms to proliferate and be transmitted. Apoptosis, a critically important host defence mechanism, contributes to the elimination of pathogen-infected cells. To oppose the immune and inflammatory responses induced by infection, many viral proteins interact with apoptotic signals to regulate apoptosis. Apoptosis inhibition is essential for the maintenance of latent infection by herpesvirus. Understanding the anti-apoptotic mechanisms of herpesvirus will greatly improve the ability to develop new drugs and vaccines for treatment and prevention; these drugs could inhibit virus release, adjust latency to reduce viral infection or promote the death of infected cells in the early stage of infection.

Disrupting apoptosis is an effective strategy for virus replication and dissemination, but the apoptotic signalling pathway of the host cells is not isolated; interplay exists with other cellular defence mechanisms. Because of the co-evolution of the virus and the host, viral anti-apoptotic factors have evolved to exploit multiple immune pathways, including the autophagy, IFN and NF *κ*B signalling pathways.^153, 154^ Interestingly, target diversity does not depend on the anti-apoptotic properties of the virus.^155^ Therefore, studies that specifically clarify how viruses inhibit apoptosis or interact with other signalling networks to promote replication will potentially lead to improved herpesvirus treatment.

## Figures and Tables

**Table 1:** *α*-Herpesvirus gene or protein that negatively modulates the apoptosis pathway

**Virus gene or protein**	**Location in virus, expression kinetics**	**Cellular target for viral protein**	**Effect of modulating apoptotic pathway**	**Representative references**
LAT	Latency	Caspase-3/9, AKT, c-FLIP, NF*κ*B	Intrinsic pathway, extrinsic pathway	^70, 73, 76^
ICP22	Lytic, IE(*α*)	Caspase-3, p53, eIF-2*α*, viral gene	Intrinsic pathway	^86, 88, 93^
ICP27	Lytic, IE(*α*)	NF-*κ*B, JNK, p38, Bcl-2 family	Intrinsic pathway, extrinsic pathway	^101, 106, 111^
US3	Tegument, L(*γ*)	Caspase-3, Bcl-2 family, NF-*κ*B, AKT, TLR2, PAK, PDCD4	Intrinsic pathway, extrinsic pathway	^125, 129, 132, 133, 142^
US5(gJ)	Envelope, L(*γ*)	Caspase-3, Caspase-8, F_0_F_1_ ATP synthase	Extrinsic pathway	^85, 120^
US6(gD)	Envelope, L(*γ*)	NF-*κ*B, HVEM	Extrinsic pathway	^149, 151^
US8(gE)	Envelope, L(*γ*)	Bim	Intrinsic pathway	^152^

Abbreviation: HVEM, herpesvirus entry mediator.
